# Synthesis and Characterization of Tetracycline Loaded Methionine-Coated NiFe_2_O_4_ Nanoparticles for Anticancer and Antibacterial Applications

**DOI:** 10.3390/nano12132286

**Published:** 2022-07-03

**Authors:** Faten Eshrati Yeganeh, Amir Eshrati Yeganeh, Bahareh Farasati Far, Afsoun Mansouri, Belay Zeleke Sibuh, Saravanan Krishnan, Soumya Pandit, Walaa F. Alsanie, Vijay Kumar Thakur, Piyush Kumar Gupta

**Affiliations:** 1 Department of Chemistry, Science and Research Branch, Islamic Azad University, Tehran 1477893855, Iran; ffyeganeh@gmail.com; 2 Department of Microbiology, Noor Dahesh Institute of Higher Education, Meymeh 45789427600, Iran; amireshratiyegane@gmail.com; 3 Department of Chemistry, Iran University of Science and Technology, Tehran 1684613114, Iran; bahar.ferasati@gmail.com; 4 School of Pharmacy and Pharmaceutical Sciences, Tehran Medical Sciences, Islamic Azad University, Tehran 1477893855, Iran; afsoonmansori@yahoo.com; 5 Department of Biotechnology, School of Engineering and Technology, Sharda University, Plot no. 32–34, Knowledge Park III, Greater Noida 201310, Uttar Pradesh, India; belayzeleke63@yahoo.com; 6 Creative Carbon Labs Pvt. Ltd., Chennai 600113, Tamil Nadu, India; sara.krish87@gmail.com; 7 Department of Life Sciences, School of Basic Sciences and Research, Sharda University, Plot no. 32–34, Knowledge Park III, Greater Noida 201310, Uttar Pradesh, India; sounip@gmail.com; 8 Department of Clinical Laboratories Sciences, The Faculty of Applied Medical Sciences, Taif University, Taif 21944, Saudi Arabia; w.alsanie@tu.edu.sa; 9 Biorefining and Advanced Materials Research Centre, Scotland’s Rural College (SRUC), Kings Buildings, Edinburgh EH9 3JG, UK; 10 School of Engineering, University of Petroleum & Energy Studies (UPES), Dehradun 248007, Uttarakhand, India; 11 Centre for Research & Developments, Chandigarh University, Mohali 140413, Punjab, India; 12 Department of Biotechnology, Graphic Era Deemed to be University, Dehradun 248002, Uttarakhand, India

**Keywords:** methionine-NiFe_2_O_4_ nanoparticles, drug release, antibacterial, anticancer, cytotoxicity

## Abstract

In the present study, nickel ferrite (NiFe_2_O_4_)-based smart magnetic nanoparticles were fabricated and coated with methionine. Physiochemical characterization of the obtained Met-NiFe_2_O_4_ nanoparticles revealed the presence of methionine coating over the nanoparticle surface. Drug release study indicated that Tet-Met-NiFe_2_O_4_ nanoparticles possess pH-responsive controlled drug release behavior for tetracycline (Tet). The drug loading content for Tet was found to be 0.27 mg/L of nanoparticles. In vitro cytotoxicity test showed that the Met-NiFe_2_O_4_ nanoparticles is biocompatible. Moreover, this magnetic nanostructured material shown strong anticancer property as these nanomaterials significantly reduced the viability of A375 cells when compared to free Tet solution. In addition, Tet-Met-NiFe_2_O_4_ nanoparticles also showed strong antibacterial activity against different bacterial pathogens.

## 1. Introduction

In today’s world, hospital-acquired infections caused by viral, bacterial, and fungal pathogens remain the primary concern and biggest challenge among the healthcare workers [1,2,3,4,5]. Antimicrobial molecules include antibiotics and biocides having a bactericidal/bacteriostatic effect on bacteria. Antibiotic is an active substance of synthetic or natural origin which is used to eradicate bacterial infections in humans or animals. While biocide is an active chemical molecule to control the growth of or kill bacteria. Hence, antimicrobial activity exhibits an inhibitory or lethal effect of a biocidal product or an antibiotic [6,7,8,9]. Biocides and antibiotics may share some common behavior and properties in their respective activity and in the resistance mechanisms developed by bacteria. Today, it is important to weigh the risks of selecting antibiotic resistant bacteria by biocide use correctly and to have a clear view of the corresponding emerging health risk. Moreover, understanding the selection and dissemination of biocide resistant pathogens is very important for combating the dissemination of health care associated diseases and foodborne pathogens.

Antibiotics are broadly classified as antibacterial, antifungal, or antiviral drugs depending on their target group [6,7,8]. For many decades, antibiotics are widely utilized in medical procedures ranging from organ transplants to chemotherapy [10]. From this, the significance of antibiotics is understood as it protects the host cells from microbial or viral infections [11,12]. Towards this, an antibiotic, Tetracycline (Tet), has been extensively used to treat various bacterial infections [13,14]. In addition, Tet is a well-recognized antibiotic used in the treatment of skin cancer and shown to inhibit several other cancer types.

The functional role of antibiotics is mainly based on the drug delivery system and the mechanism of their controlled delivery to targeted affected cells [15,16,17,18]. Recently, several organic-inorganic hybrid nanoparticles have been extensively used in anticancer therapy. Indeed, methionine is one of the essential amino acids i.e., required for the human growth due to its antioxidant activity. The activated functional groups of methionine (-COOH and -NH_2_) are used to conjugate the metal atoms, and further the loading and release behavior are investigated. The design and synthesis of organic–inorganic hybrid nanoparticles through combining the advantages of both organic and inorganic counterparts may improve the overall properties, such as particle size, surface charge, and many other physicochemical properties [19,20]. Roca et al. (2012) reported that modified magnetic NPs (MNPs) has shown increased efficacy under in vivo conditions [21].

Several recent findings have emphasized the therapeutic role of MNPs in treating the disease [22,23,24]. Nanoparticles whose particle size ranging between 10–100 nm have shown properties to improve drug bioavailability and facilitate the targeted accumulation of drugs inside the cell through enhanced permeability and retention (EPR) effect [25,26]. Magnetic spinel ferrite nanoparticles are widely used in biotechnology. The main advantage of magnetic nanoparticles is that can be readily isolated from the solution media using an external magnetic field. NiFe_2_O_4_ nanoparticles have gained worldwide acceptance due to their low coercivity, high saturation magnetization, excellent chemical stability, high Curie temperature, and electromagnetic performance [27]. However, to the authors’ best knowledge, the utilization of NiFe_2_O_4_ magnetic nanoparticles in the field of biomedicine is available. Further, NiFe_2_O_4_ nanoparticles are widely used as an in vivo magnetic hyperthermia agent in biomedicine [28]. NiFe_2_O_4_ nanoparticles exhibited an inverse spinel structure with Ni^+2^ in octahedral sites (Ni(OH)) and Fe^+3^ equally distributed between tetrahedral (Fe (Td)) and octahedral sites (Fe(OH)) of the O^2−^ FCC (Face-Centered Cubic) cell [29]. The complete crystalline structure belongs to $O_{h}^{7}$ space group with oxygen atoms occupying the 32e positions, Fe (Td) atoms occupying the 8a ones and the Ni(OH) and Fe(OH) atoms are distributed on 16d positions [30]. Different methods, including solvothermal, sol-gel, co-precipitation, microemulsion, and thermal decomposition, have been utilized to fabricate magnetic nanostructures of varying morphologies and structural variants [31,32,33,34] which has immense scope and applications in biomedical science.

Various amino acids have been successfully applied in the preparation of magnetic nanoparticles. However, the use of methionine in the fabrication of nickel-ferrite based nanocomposite is not reported yet. Recently, Saykova et al. synthesized magnetic NiFe_2_O_4_@Au crystal nanoparticles using the amino acid methionine as a reducing and stabilizing agent [35]. The nanoparticles obtained in this study after three stages of gold deposition had an average particle size of about 120 nm, which is relatively large for cellular adsorption studies and for biomedical applications thereof. In this study, methionine-coated nickel ferrite nanoparticles with an average particle size of about 27 nm are synthesized in a simple and cost-effective step by reflux method, which is the preferred magnetic nanomaterial for various biological applications.

Recently, non-coated nickel ferrite magnetic nanoparticles were synthesized by Majed et al. and tested in a rat model [36]. However, tetracycline loading on this nanostructure (Met-NiFe_2_O_4_), and its effect on normal and A-375 cancer cells has not been studied. In addition, the loading of tetracycline on Met-NiFe_2_O_4_ as a biocompatible and targeted nanocarrier and its effect on bacterial growth remain unexplored. Compared to previous literature, it can be concluded that we have prepared nickel ferrite nanoparticles coated with methionine in a facile synthetic route which is explored for anticancer and antibacterial applications.

In the present study, a simple and effective strategy is developed to synthesize Met-NiFe_2_O_4_ nanoparticles employing a one-step reflux procedure. Various characterization studies are performed to examine the shape, structure, and magnetic characteristics of Met-NiFe_2_O_4_ nanoparticles. Tet antibiotic is used to explore the drug loading and release profile of Met-NiFe_2_O_4_ nanoparticles, for the first time. Moreover, MTT tests are used to assess the in vitro cytotoxicity of Met-NiFe_2_O_4_ nanoparticles before and after Tet loading of varying doses and incubation periods. Furthermore, antibacterial activity of Met-NiFe_2_O_4_ nanoparticles against pathogenic bacteria is assessed. To date, the cytotoxicity and antibacterial properties of Met-NiFe_2_O_4_ nanoparticles have not been reported.

## 2. Materials and Methods

### 2.1. Materials

Ferric chloride hexahydrate (FeCl_3_·6H_2_O), Nickel chloride hexahydrate (NiCl_2_·6H_2_O), sodium hydroxide, methionine, and other chemicals were procured from Merck, Germany. A-375 and HFF cells were obtained from the Pasteur cell bank. *Staphylococcus aureus* (ATCC 23235), *Escherichia coli* (ATCC 25922), *Pseudomonas aeruginosa* (ATCC 15442), and *Enterococcus faecalis* (ATCC 29212) strains were collected from The Pasteur Institute of Iran.

### 2.2. Fabrication of Met-NiFe_2_O_4_ Nanoparticles

**Figure 1:**
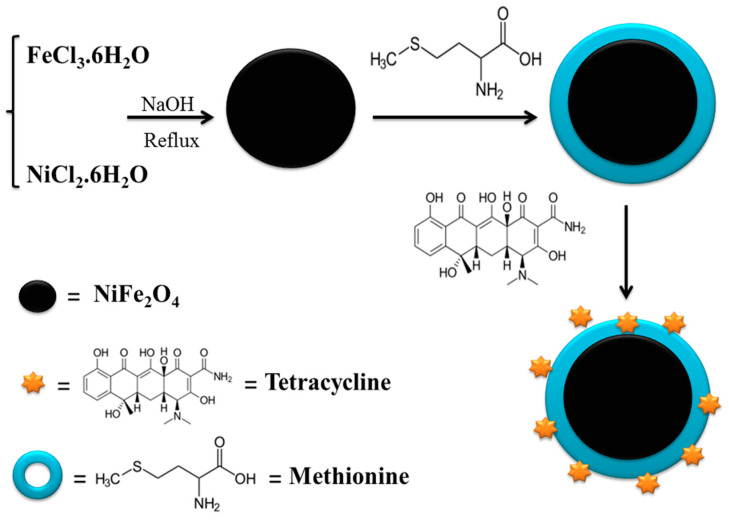
Synthesis and Tet loading on Methionine-coated nickel ferrite nanoparticles (Met-NiFe_2_O_4_ nanoparticles).

Met-NiFe_2_O_4_ nanoparticles were synthesized by reflux method (as shown in Figure 1). In brief, 2.431 g of FeCl_3_·6H_2_O and 1.069 g of NiCl_2_·6H_2_O were dissolved in 120 mL of distilled water (d.H_2_O), followed by the addition of 1.5 M NaOH solution to adjust the pH to 12. Then, a brown colored solution appeared, and was filtered and washed several times with d.H_2_O until the pH of the filtrate becomes neutral (7). Next, 1.5 g of methionine was added. The resulting mixture was heated to 70–80 °C and refluxed for 3 h. The final product was collected by strong magnetic separation and rinsed three times with absolute ethanol and d.H_2_O. In a similar way, NiFe_2_O_4_ nanoparticles were synthesized, but without methionine.

### 2.3. Physicochemical Characterization Studies

X-ray diffraction (XRD) analysis NiFe_2_O_4_ and Met-NiFe_2_O_4_ nanoparticles was carried out using the STOE STADI-P instrument (STOE, & Cie GmbH, Darmstadt, Germany). Field Emission-Scanning Electron Microscopy (FESEM) (Zeiss-EHT-10.00 kV, Carl Zeiss SMT AG Company, Oberkochen, Germany) and High Resolution-Transmission Electron Microscopy (HR-TEM) (Zeiss-EM10C-100 kV, Carl Zeiss SMT AG Company, Oberkochen, Germany) instruments were used to determine morphology and particle size of nanoparticles respectively. The particle size was measured in a dry state through the particle size distribution curve. Next, Fourier Transform Infrared Spectroscopy (FTIR) (Nexus 870, Nicolet, Madison, WI, USA) was also performed. Absorbance measurements at 275 nm through means of UV/visible spectroscopy (Shimadzu UVS-1700, Shimadzu Corporation, Kyoto, Japan) was employed to study the amount of drug adsorbed on nanoparticle surface and the controlled drug release behavior. Thermal properties were analyzed by Thermogravimetric Analyzer (TGA) (Shimadzu TA Q600, Shimadzu TA Instrument SDT Q600, New Castle, DE, USA) in a temperature range of 25 to 800 °C under Nitrogen atmosphere at a constant heating rate. Finally, the magnetic properties were studied by Quantum Design MPMS-XL-7 instrument (MPMS-XL-7), San Diego, CA, USA.

### 2.4. Average Hydrodynamic Size and Zeta Potential Measurement

The average hydrodynamic size (Z-average) (based on light diffraction), particle size distribution (PDI), and average zeta potential (based on electrophoretic movement of particles) of NiFe_2_O_4_, Met-NiFe_2_O_4_ and Tet-Met-NiFe_2_O_4_ nanoparticles were determined by Malvern Zetasizer 2000 HS instrument (Malvern, UK) where nanoparticles were diluted 100 times in distilled water at 25 ± 0.1 °C prior to experiment.

### 2.5. Tetracycline Loading and Release Behavior

Various concentrations of Tet were added to 0.2 mg of Met-NiFe_2_O_4_ nanoparticles and incubated in the dark conditions for 24 h at room temperature. The resulting Tet-Met-NiFe_2_O_4_ nanoparticles were pelleted using centrifuge, and the collected supernatant was subjected to absorbance measurements at 275 nm using UV/Visible spectroscopy to determine the drug loading efficiency of Tet (Figure 1).

Next, 10 mg of Tet-Met-NiFe_2_O_4_ nanoparticles was suspended into 10 mL of PBS buffer of pH 5 and 7.4 at 37 °C under dark conditions. Then, 1 mL supernatant was collected at different time intervals and replaced with fresh PBS. Finally, the concentration of released Tet was calculated using UV/Visible spectroscopy (Shimadzu UVS-1700, Shimadzu Corporation, Kyoto, Japan). The drug release data were fitted with different kinetic models’ equations for determining the mechanism of drug release from Tet-Met-NiFe_2_O_4_ nanoformulations. The most appropriate drug release kinetic model was determined by analyzing the regression coefficients of graphs. According to this, the drug release mechanism was determined from the drug delivery system.

### 2.6. In Vitro Cytotoxicity

A standard MTT test was performed to analyze the in vitro cytotoxicity of Tet-Met-NiFe_2_O_4_ nanoparticles on A375 and HFF cell lines. These cell lines were cultured using RPMI-1640 fresh medium supplemented with 10% FBS and 1% penicillin/streptomycin in a humid CO_2_ incubator with 5% carbon dioxide at 37 °C. Once the cells had achieved 85–95% confluence, the media was aspirated. Detachment of the cell monolayer was performed using trypsin-EDTA (0.25% (*w*/*v*)). Then, the detached cells were resuspended in a complete growth medium, labeled with trypan blue, and counted using hemocytometer. Different concentrations of Tet-Met-NiFe_2_O_4_ (0–70 μg/mL) were added to the cultured A375 and HFF cells and incubated for 72 h. For cell proliferation studies, the cells were incubated with 0.5 mg/mL of MTT reagent for 4 h. Then, the purple crystal formazan formed was dissolved in 100 μL of DMSO for the colorimetric determination of the cells’ oxidoreductase enzymatic activity. The absorbance values of both control and test concentrations were measured at 570 nm while reference blank measurements taken at 630 nm, and then the cell viability and IC_50_ values were calculated [7,15,37]. The percentage of cell viability was calculated using Equation (1).

(1) $$Cell\;Viability\;\left(\%\right)=\frac{absorbance\;of\;treated\;cells}{absorbance\;of\;control\;cells}\times 100$$

### 2.7. Antibacterial Activity

The antibacterial activities of free Tetracycline and Tet-Met-NiFe_2_O_4_ nanoparticles were evaluated against both Gram-positive and Gram-negative bacteria, such as *E. coli*, *P. aeruginosa*, *S. aureus*, and *E. faecalis*, using microdilution method. Next, the obtained data was used to calculate the minimum bactericidal concentrations (MBC) and minimum inhibitory concentrations (MIC). Different samples were initially made using Mueller–Hinton Broth (MHB), then 100 μL of each sample was added into 96-well microplates. Approximately 0.5 McFarland standard bacterial suspension was prepared and diluted in Mueller–Hinton Broth to achieve a final concentration of 1 × 10^6^ colony forming units (CFU)/mL. Then, 100 µL of diluted bacterial suspension was mixed with 100 µL of samples (with different concentrations as mentioned above) in 96-well microplates and incubated at 37 °C for 18 h. Bacterial suspension without the drug acts as positive control, while the negative control was the highest drug concentration without bacteria. MBC test was performed to confirm the results of MIC test. Consequently, 100 µL of clear wells with no visible bacterial growth was transferred to petri plates containing MHA (Mueller–Hinton Agar) medium and incubated for overnight at 37 °C [7,8].

### 2.8. Statistical Analysis

All tests were performed in triplicates (*n* = 3) and each test repeated at least three independent times. The equality of variance and normality were checked by Brown–Forsythe and Shapiro–Wilk tests, respectively. Then, the data was compared using one way or two-way analysis of variance (ANOVA) with repeated measures, followed by Tukey’s or Sidak’s post hoc comparison test. The results were given as mean ± SD. The differences were considered significant when a *p*-value of less than 0.05 is considered statistically significant. * *p* < 0.05, ** *p* <0.01, *** *p* < 0.001.

## 3. Results and Discussion

### 3.1. Synthesis and Physicochemical Characterizations of Tet-Met-NiFe_2_O_4_ Nanoparticles

#### 3.1.1. XRD Analysis

**Figure 2:**
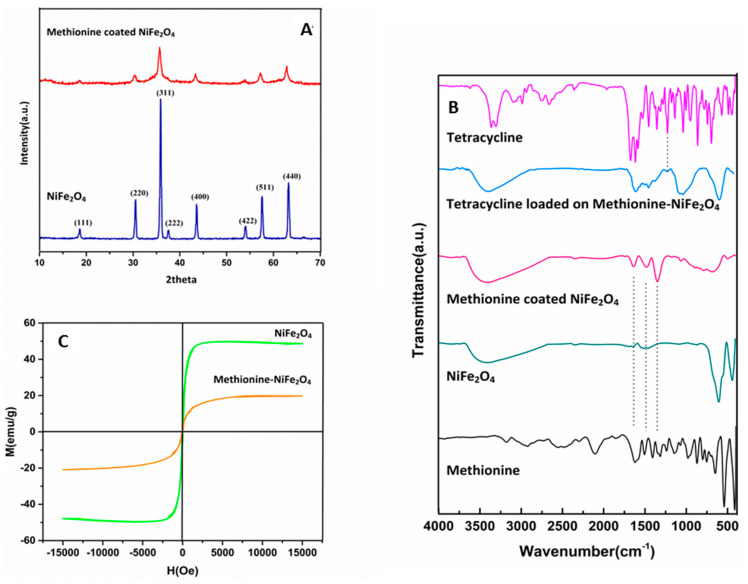
X-ray diffraction patterns of NiFe_2_O_4_ nanoparticles with and without methionine coating (**A**), FTIR spectrum of methionine, Tet, NiFe_2_O_4_ nanoparticles, Met-NiFe_2_O_4_ nanoparticles, Tet-Met-NiFe_2_O_4_ nanoparticles, (**B**) and Magnetization curves of NiFe_2_O_4_ and Met-NiFe_2_O_4_ nanoparticles (**C**).

The method of fabrication of Met-NiFe_2_O_4_ nanoparticles is outlined in Figure 1. XRD analysis showed the diffraction peaks of crystal spinel NiFe_2_O_4_ nanoparticles at 18.60°, 30.49°, 35.88°, 37.45°, 43.60°, 53.98°, 57.54°, and 63.12° 2Ɵ values (JCPDS No. 98-006-0930) while in the case of Met-NiFe_2_O_4_ nanoparticles, the diffraction peaks were observed at 18.57°, 30.24°, 35.73°, 37.44°, 43.32°, 53.69°, 57.28°, and 62.77° 2θ values (JCPDS No. 98-006-0930) (Figure 2A). The entry of methionine into the network cavity and an increase in network space is reflected through a drip in 2θ value corresponding to crystal spinel NiFe_2_O_4_ nanoparticles. The characteristic diffraction peaks for two samples are indexed to the crystal planes of (111), (220), (311), (222), (400), (422), (511), and (440). Met-NiFe_2_O_4_ nanoparticles with average size of 27 nm as calculated from the full-width value at half maximum (FWHM) of broadened characteristic peaks using Debye–Scherrer’s Equation (2).

(2) $$D=\frac{0.9\lambda }{\mathrm{FWHM}\;\cos\theta }$$

where D denotes the crystalline size, β is the full width at half maximum (FWHM), θ represents the Bragg angle corresponding to the peak, and λ is the wavelength of the X-rays.

#### 3.1.2. FT-IR Spectral Analysis

FTIR analysis was used to study the nature of functional groups present on the surface of MNPs. The FTIR spectra of methionine, free Tet, bare NiFe_2_O_4_, Met-NiFe_2_O_4_ nanoparticles, and Tet-Met-NiFe_2_O_4_ nanoparticles are shown in Figure 2B. The Methionine amino acid spectrum is a combination of carboxylate salts and the first type amine. The symmetric and asymmetric N-H bending assigned in the region at 1517 cm^−1^ and 1630 cm^−1^ respectively. Furthermore, the symmetric and asymmetrical stretching of the COO^−^ bond was exhibited at 1419 cm^−1^ and 1600 cm^−1^, respectively [38]. C–O bond represented at 1232–1330 cm^−1^. The inherent vibration of tetrahedral and octahedral metal-oxygen complexes of NiFe_2_O_4_ is ascribed to regions observed at 400 and 600 cm^−1^, respectively, which are mostly dependent on Fe–O distance [38].

FTIR spectrum of Met-NiFe_2_O_4_ nanoparticles revealed that methionine was present on the surface of NiFe_2_O_4_ nanoparticles. Tet features two small vibration at 500–569 cm^−1^ in its FT-IR spectra, which is linked to out-of-plane ring deformation. The regions at 1000–1257 cm^−1^ are associated with C–H in-plane deformation vibrations, while those at 998 cm^−1^ are associated with C–N stretching. Two bands appeared at 1462 and 1366 cm^−1^ displaying the bending vibrations of C–H and CH_3_ groups, respectively. Band of C=C stretching is observed at 1571–1632 cm^−1^. C–H and CH_3_ stretching are attributed to the vibrational at 3013–3078 cm^−1^ and 2819–2949 cm^−1^. Bands of N-H and O-H stretching is ascribed to the regions which seen at 3319–3340 cm^−1^ [39]. After Tet loading, the regions at 998–1257 cm^−1^ were originated in the FTIR spectrum, which is most likely due to C–N stretching and C–H vibration bands in-plane deformation of Tet. These findings supported that methionine’s gets effectively coated onto MNPs, and, thus, possess good potential for drug loading and acts as smart nanocarrier for drug delivery.

#### 3.1.3. Magnetic Property Analysis

Figure 2C displayed the magnetic properties of bare NiFe_2_O_4_ and Met-NiFe_2_O_4_ nanoparticles at room temperature in a magnetic field ranging from −15 kOe to 15 kOe. These MNPs exhibited the superparamagnetic behavior. The saturation magnetization (Ms) of bare NiFe_2_O_4_ and Met-NiFe_2_O_4_ nanoparticles were calculated to be 47.56 and 19.80 emu/g, respectively. Further, the saturation magnetization (Ms) of Met-NiFe_2_O_4_ nanoparticles was also reduced at room temperature. Superparamagnetic behavior is an important feature for biomaterials, which facilitate their tracking in the magnetic field while keeping the advantage of a stable and homogeneous suspension during drug delivery.

#### 3.1.4. Size and Morphology Analysis

**Figure 3:**
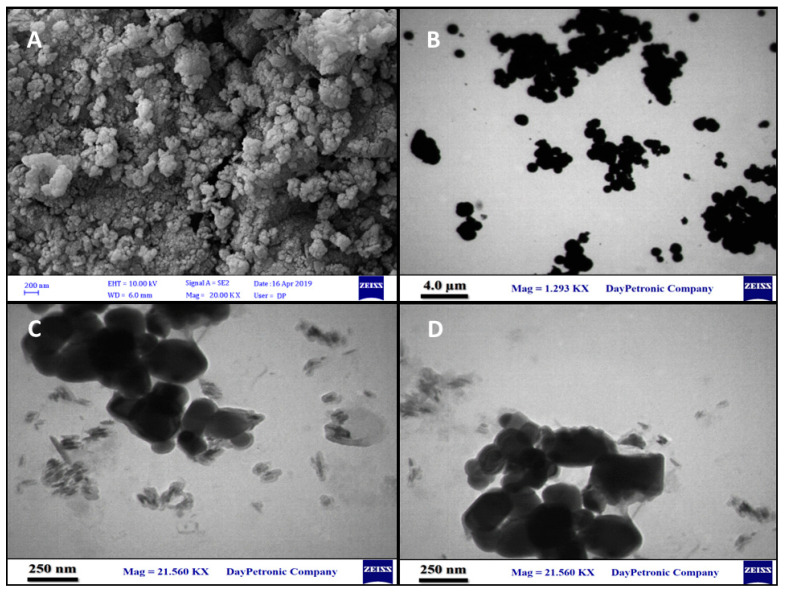
FE-SEM of Met-NiFe_2_O_4_ nanoparticles (**A**), TEM images of NiFe_2_O_4_ nanoparticles (**B**), Met-NiFe_2_O_4_ nanoparticles (**C**), and Tet-Met-NiFe_2_O_4_ nanoparticles (**D**).

FE-SEM (Figure 3A) and HR-TEM (Figure 3B,C) images indicated that NiFe_2_O_4_ nanoparticles_,_ Met-NiFe_2_O_4_ nanoparticles, and Tet-Met-NiFe_2_O_4_ nanoparticles, were found to be spherical in shape and possess nearly uniform sizes. Besides this, the TEM images of Met-NiFe_2_O_4_ and Tet-Met-NiFe_2_O_4_ nanoparticles showed a mild aggregation, probably due to the strong magnetic interactions between the NiFe_2_O_4_ nanoparticles. Methionine was successfully coated onto the surface of NiFe_2_O_4_ nanoparticles, and the particle size was calculated to be 28–29 nm.

#### 3.1.5. Thermogravimetric Analysis

**Figure 4:**
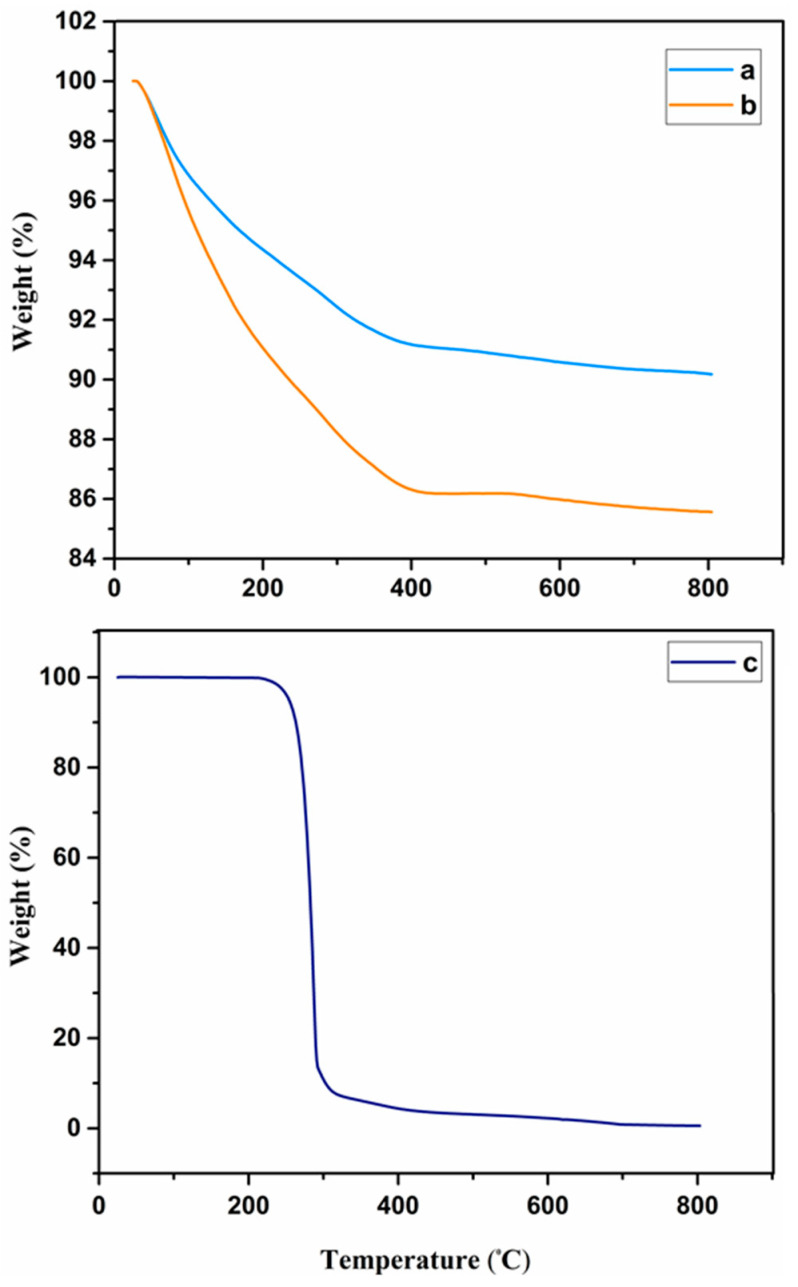
Thermogravimetric curves of NiFe_2_O_4_ nanoparticles (**a**), Met-NiFe_2_O_4_ nanoparticles (**b**), and methionine (**c**).

Thermogravimetric analysis (TGA) of methionine, NiFe_2_O_4_ nanoparticles, and Met-NiFe_2_O_4_ nanoparticles (Figure 4) were recorded. TGA profile showed that the initial weight loss observed at 150 to 260 ℃ could be due to thermal decomposition of carbon dioxide, water and fine molecules trapped between the layers. On the other hand, heat-induced thermal degradation of methionine could be noticed at 260–300 ℃, which is also consistent with TGA profile of amino acid, methionine. Further increase in temperature above 350 ℃ showed no change in weight which could be due to the high stability of the nanostructure [33,40]. The weight loss observed near 300 °C for NiFe_2_O_4_ nanoparticles and Met-NiFe_2_O_4_ nanoparticles were found to be 8.3% and 13.8%, respectively. Difference in the weight loss between NiFe_2_O_4_ nanoparticles and Met-NiFe_2_O_4_ nanoparticles near 300 °C indicated that methionine was successfully coated onto the surface of the NiFe_2_O_4_ nanoparticles.

### 3.2. Size Distribution and Zeta Potential Measurement

**Figure 5:**
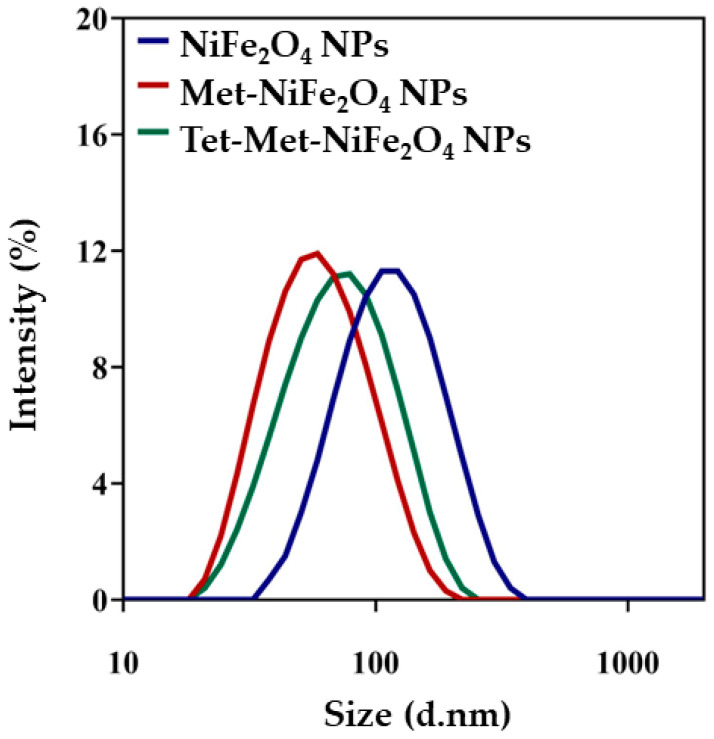
The particle hydrodynamic size distribution curves of NiFe_2_O_4_, Met- NiFe_2_O_4_, and Tet-Met-NiFe_2_O_4_ nanoparticles.

The average hydrodynamic size, particle size distribution, and average zeta potential of all nanoparticles were measured at the same concentration and pH values. The particle hydrodynamic size distribution graph was plotted using Gaussian theorem for NiFe_2_O_4_, Met-NiFe_2_O_4_, and Tet-Met-NiFe_2_O_4_ nanoparticles (Figure 5). All the calculated values are given in a tabulated form (Table 1).

### 3.3. Drug Loading and Release Study

**Figure 6:**
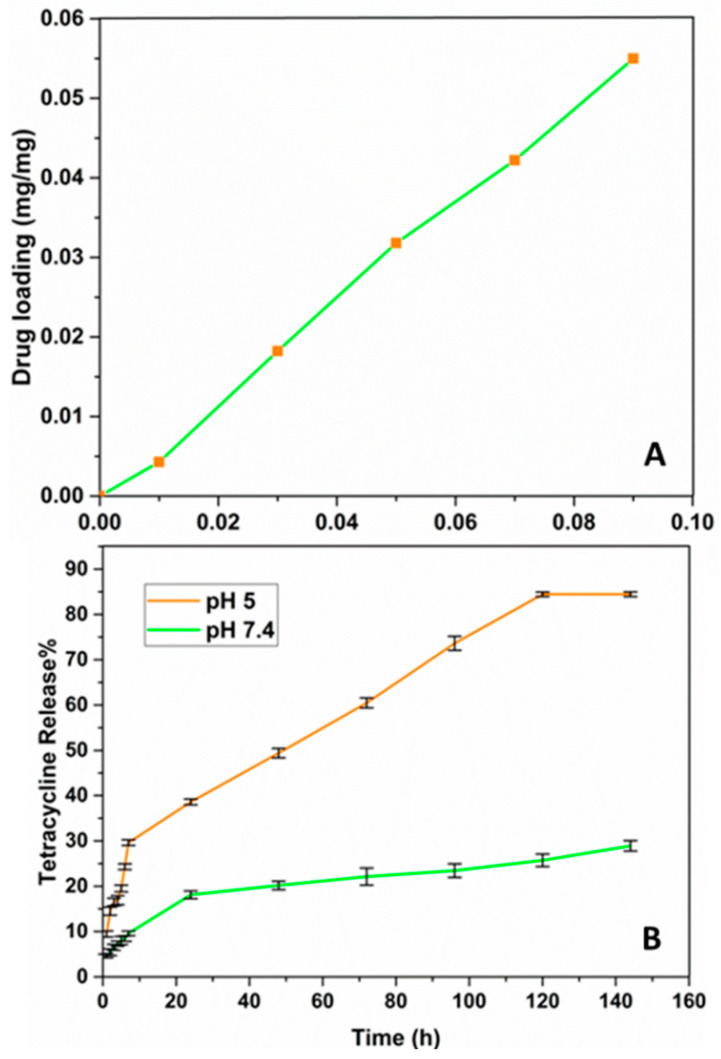
Tet loading on Met-NiFe_2_O_4_ nanoparticles (**A**) and Tet release from Met-NiFe_2_O_4_ nanoparticles at different pH values (**B**).

Tet was further loaded onto Met-NiFe_2_O_4_ nanoparticles, and the drug loading and release profile was assessed. As shown in Figure 6A, the maximum loading capacity of Met-NiFe_2_O_4_ nanoparticles reached 0.055 mg/mg, when the initial Tet concentration was 0.09 mg/mL. In other words, 0.27 mg of Tet was loaded onto 1 mg of nanocarrier. The amount of drug loading is enhanced through increasing the initial drug concentration taken for the study. This is possible due to the larger surface area and hydrogen bonding between Tet and Met-NiFe_2_O_4_ nanoparticles. To evaluate the drug release profile, the Tet-Met-NiFe_2_O_4_ nanoparticles were suspended in the buffer media of pH 5 and 7.4. Figure 6B shows the pH-dependent release of Tet from Met-NiFe_2_O_4_ nanoparticles. Drug release was observed at pH values near the tumor’s environment (pH 5), while the lower drug release was detected at pH 7.4. The pH-sensitive drug release of the nanocarrier under neutral conditions (pH 7.4) could reduce the drug loss during blood transportation and lessen the side effects of anti-cancer drugs observed in normal cells. In the simulated environment of tumors (pH 5), this above-mentioned property of nanomaterial facilitates efficient anti-cancer drug release [41]. The pH-responsive nano-based delivery tools could lead to site-specific release of therapeutic cargos through cleaving pH-sensitive bonds across the pH gradient and augment an increase in toxicity under acidic conditions prevalent in the tumor regions [42,43].

**Figure 7:**
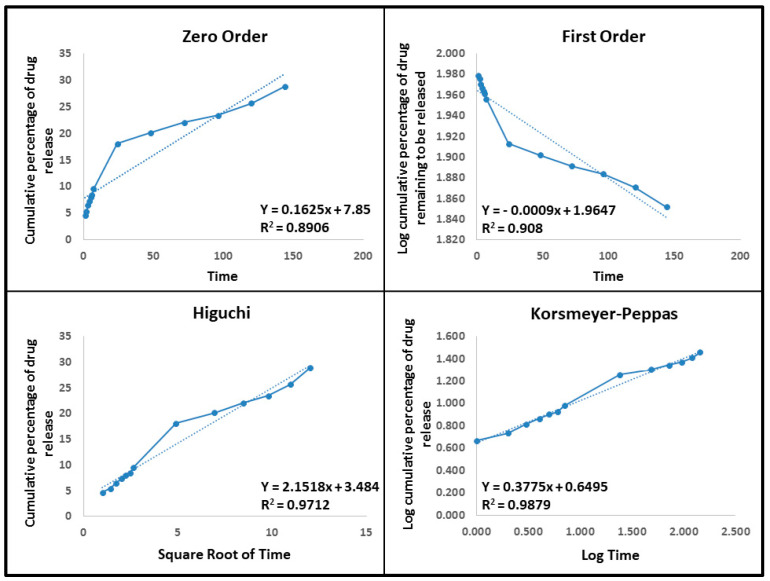
Drug release kinetic models like zero-order, first-order, Higuchi, and Korsmeyer–Peppas models used to study the tetracycline release from Tet-Met-NiFe_2_O_4_ nanoparticles at pH 7.4.

**Figure 8:**
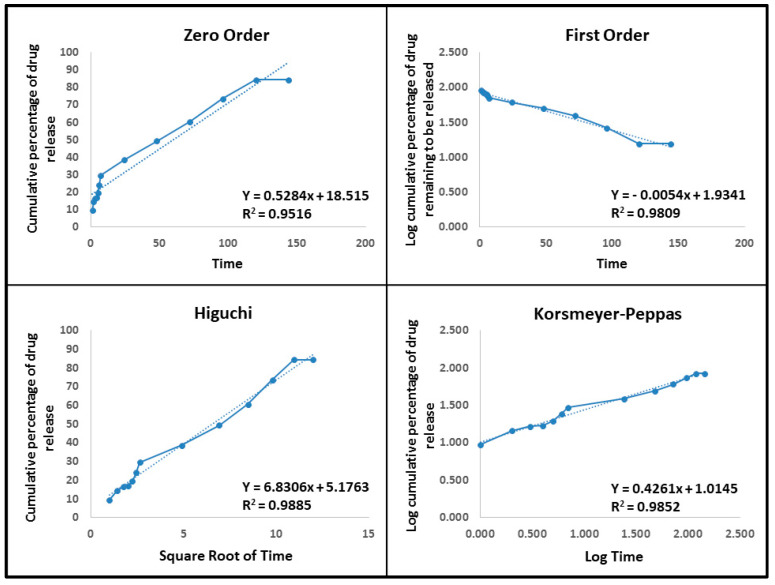
Drug release kinetic models like zero-order, first-order, Higuchi, and Korsmeyer–Peppas models used to study the tetracycline release from Tet-Met-NiFe_2_O_4_ nanoparticles at pH—5.

At neutral and acidic pH, the release kinetics of drug-loaded Met-NiFe_2_O_4_ nanoparticles were assessed using zero-order, first-order, Higuchi, and Korsmeyer–Peppas models, as shown in Table 2. An ideal kinetic model with a higher linear regression coefficient (close to 1) and determination (*R*^2^) describe the drug release mechanism in the best way. The Higuchi model and Korsmeyer–Peppas model described the release kinetics at pH 5 and pH 7.4 (*n* < 0.45), whereas the Korsmeyer–Peppas model indicated the Fickian diffusion mechanism. It is worth mentioning that the drug gets distributed through diffusion in these cases (Higuchi model and Fickian diffusion mechanism). The pH-dependent drug release is well suited for cancer therapy, where the cancerous cells have an acidic intercellular environment while the healthy cells do not. The mathematical analysis is illustrated at pH 7.4 and pH 5 in Figure 7 and Figure 8.

### 3.4. Cytotoxicity Studies

**Figure 9:**
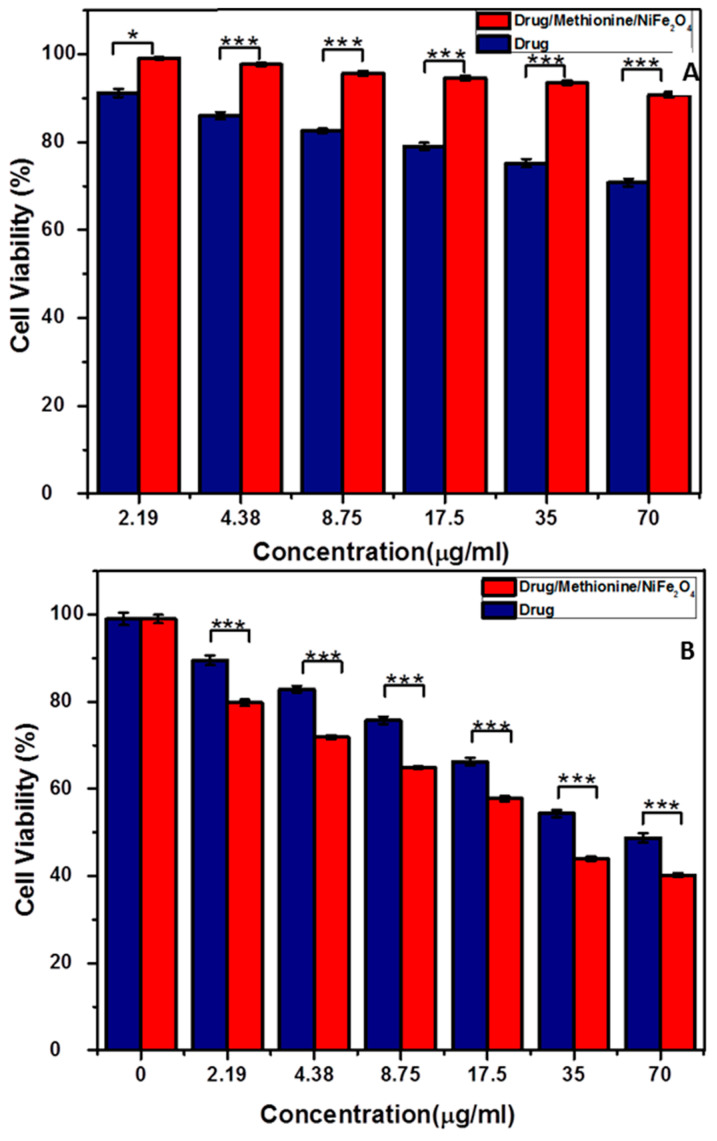
Cell viability of Tet-Met-NiFe_2_O_4_ nanoparticles and free Tet solution after 72 h incubation with (**A**) HFF cells and (**B**) A375 cells. The data is expressed as mean ± SD (*n* = 5). * *p* < 0.05, *** *p* < 0.001.

The cytotoxicity of Tet-Met-NiFe_2_O_4_ nanoparticles and free Tet were investigated using MTT test thrice for the concentration range of 0–70 μg/mL. Melanoma (A375 cells) and Human Foreskin Fibroblasts (HFF cells) were selected for the MTT assay. Results indicated that both free Tet and Tet-Met-NiFe_2_O_4_ nanoparticles showed cytotoxicity on cancer cells while toxicity was not observed with normal cells. As shown in Figure 9B, the cytotoxicity of Tet-Met-NiFe_2_O_4_ nanoparticles (IC_50_ = 32.55 ± 7.31 μg/mL) was higher than free Tet (IC_50_ = 76.58 ± 5.78 μg/mL) at 72 h. One possible reason for the differences in toxicity is due to difference in intracellular uptake of drug between the free drug and drug-loaded nanoparticles. More importantly, when the free drug is taken by cells, its release is faster, which means it is available to cell in less time owing to cross-sectional effect. When the drug is loaded onto nanoparticles, it exhibits controlled release, sustained effect, and as a result, the overall therapeutic efficacy is significantly improved [22,44]. Furthermore, Tet-Met-NiFe_2_O_4_ nanoparticles displayed no cytotoxicity on HFF cells (Figure 9A) due to excellent biocompatibility observed through coating the MNPs with amino acid methionine. These results finally showed that the biocompatible Tet-Met-NiFe_2_O_4_ nanoparticles synergistically improved the growth inhibitory effect on cancer cells.

### 3.5. Antibacterial Activity

Microdilution method was used to evaluate the antibacterial activity of free Tet and Tet-Met-NiFe_2_O_4_ nanoparticles. Table 3 showed that MIC of Tet-Met-NiFe_2_O_4_ nanoparticles against pathogenic bacteria were between 1 to 8 µg/mL. However, Tet-Met-NiFe_2_O_4_ nanoparticles had potent antibacterial activity against Gram-negative bacteria with a significant reduction in MIC and MBC values i.e., 2–3-folds as compared to that of free Tet (Table 3). This was probably due to more penetration of Tet-Met-NiFe_2_O_4_ nanoparticles into Gram-negative bacterial cells [45]. In fact, Gram-negative bacteria possess very thin peptidoglycan layer than Gram-positive bacteria, and this structure may facilitate the penetration of nanoparticles into bacterial cells [46,47,48,49]. These data supported the idea that Met-NiFe_2_O_4_ nanoparticles can be utilized as carriers of antibiotics for antibacterial applications. Another probable antibacterial mechanism of Met-NiFe_2_O_4_ nanoparticles could be due to enhanced bacterial outer membrane permeability, which is due to the interaction of nanoparticles with the bacterial cell walls, which, in turn, alters the intrinsic membrane potential [50].

## 4. Conclusions

MNPs have been broadly explored for the development of drug delivery systems in recent years. In this study, Tet-Met-NiFe_2_O_4_ nanoparticles was fabricated for cancer therapeutics. Results suggested that methionine coating onto magnetic NiFe_2_O_4_ nanoparticles stabilize the nanoparticles and, thus, lead to more effective drug loading. Evidently, Tet was released rapidly into the cancer cells. In vitro cytotoxicity test indicated the excellent biocompatibility of magnetic nanocarriers due to the surface coating with methionine. When Met-NiFe_2_O_4_ nanoparticles were tested, no cytotoxicity was observed on both cell lines. Tet-Met-NiFe_2_O_4_ nanoparticles showed significant cytotoxicity on A375 cells, even higher than free Tet solution.

Furthermore, strong bactericidal activity was seen, when Tet-Met-NiFe_2_O_4_ nanoparticles was tested against different Gram-positive and Gram-negative bacterial pathogens. Collectively, these results presented that Met-NiFe_2_O_4_ nanoparticles like biocompatible nanocarriers could be potentially utilized for treating bacterial infections and cancer.

## Figures and Tables

**Table 1:** Average hydrodynamic Size, PDI, and average zeta potential values of the fabricated formulations.

Formulation	Size (d.nm)	PDI	Zeta Potential (mV)
NiFe_2_O_4_	168.5 ± 11.8	0.346 ± 0.02	−32.4 + 2.4
Met-NiFe_2_O_4_	72.4 ± 8.8	0.247 ± 0.06	−27.7 ± 4.3
Tet-Met-NiFe_2_O_4_	90.9 ± 10.6	0.323 + 0.02	−31.6 ± 1.2

**Table 2:** The drug release kinetic models and the parameters obtained for optimum nanoformulation.

Kinetic Models	Equation	*R* ^2^	
		Met-NiFe_2_O_4_ Nanoparticles (pH—7.4)	Met-NiFe_2_O_4_ Nanoparticles (pH—5)
Zero-Order	C_t_ = C_0_ + K_0_t	0.8906	0.9516
First-Order	LogC = LogC_0_ + K_t_/2.303	0.9080	0.9809
Higuchi	$Q=K_{h}\surd t$	0.9712	0.9885
Korsmeyer-Peppas	M_t_/M_ꝏ_ = Kt^n^	0.9879 (*n = 0.3775)	0.9852 (*n = 0.4261)

* Diffusion or release exponent.

**Table 3:** Antibacterial activity of free Tet and Tet-Met-NiFe_2_O_4_ nanoparticles.

Bacteria		*E. coli*	*P. aeruginosa*	*S. aureus*	*E. faecalis*
MIC (µg/mL)	Free Tet	8	8	8	8
	Tet-met-NiFe_2_O_4_	1	2	4	4
MBC (µg/mL)	Free Tet	16	16	16	16
	Tet-met-NiFe_2_O_4_	2	2	8	8

## Data Availability

Not applicable.
